# Best practices for recruitment in veterinary clinical trials

**DOI:** 10.3389/fvets.2024.1418747

**Published:** 2024-07-17

**Authors:** Mindy Quigley, Charly McKenna, Tracy L. Webb

**Affiliations:** ^1^Veterinary Clinical Research Office, Virginia-Maryland College of Veterinary Medicine, Virginia Tech, Blacksburg, VA, United States; ^2^Clinical Studies, Department of Clinical Sciences, Ontario Veterinary College, University of Guelph, Guelph, ON, Canada; ^3^Department of Clinical Sciences, College of Veterinary Medicine and Biomedical Sciences, Colorado State University, Fort Collins, CO, United States

**Keywords:** study recruitment, clinical trial, veterinary, incentive, retention, advertising, study design

## Abstract

A successful clinical trial requires participants, but many factors can impede effective study recruitment. To better recruit for quality veterinary clinical trials in client-owned animals that lead to improved evidence-based patient care and outcomes, there is a collective need to share and implement current best practices for recruitment strategies. These strategies should utilize a holistic view of recruitment, encompassing study design and logistics, representative participation, incentives, personnel resources, advertising, and participant retention. Although human clinical trial data and resources can provide guidance, effort also needs to be put into evaluating current practices and opportunities for process improvement that are specific to the conduct of veterinary clinical trials. Considering the power of pets as naturally occurring models of disease and as sentinels, improved conduct of veterinary clinical research has the potential to inform human health outcomes. Continued development of collaborations surrounding best practices and training opportunities in veterinary clinical research will improve the impact of veterinary clinical trials teams, while also promoting workforce development and alternate career paths for veterinary professionals.

## 1 Introduction

Clinical trials in human and veterinary medicine aim to improve patient care and address unmet therapeutic needs. Not only can such research benefit patients directly, but the similarities between naturally occurring disease processes in companion animals and humans, along with our shared exposures, mean that new therapeutics and devices evaluated for one species may have benefits for others (1). While the potential advantages of such research are clear, how we conduct clinical trials is an area of continued discussion. The veterinary research community has made strides toward a more systematic and rigorous approach to best practices in companion animal clinical studies’ design, conduct, and ethical underpinnings (2). These best practice recommendations often mirror, to the extent practicable, those for human clinical research (3). However, the literature regarding one critical aspect, recruiting veterinary patients into trials, is sparse.

Experienced clinical investigators are often painfully aware that recruitment is more complex than putting up a flier and waiting for the phone to ring or an email to arrive. The facilitation of patient enrollment through outreach efforts such as advertisements, websites, print materials, and conversations between pet owners, referring veterinarians, in-hospital colleagues and coworkers, and study personnel is crucial. Additionally, a successful recruitment strategy must encompass all phases of the study, from design through data collection, and continue even after the study closes. This narrative review discusses current information and methods of optimizing patient enrollment, including designing recruitable studies, using incentives, managing a study communication plan, and building long-term, trusting relationships with pet owners, in-hospital colleagues and coworkers, and referring veterinarians. The current landscape regarding veterinary patient recruitment and the shortcomings of some common recruitment strategies are presented along with tools and additional considerations for clinical researchers as they plan and perform this crucial aspect of conducting quality clinical research.

## 2 Why is recruitment important?

In human clinical trials, enrolling eligible participants has been cited as the most substantial workload component (4, 5), with recruitment accounting for over 30% of study costs (6). Slow or ineffective study recruitment results in lost revenue, estimated at up to $8 million USD per day of delay in pharmaceutical trials (4–7). It has been reported that 79–90% of human clinical trials incur setbacks related to patient recruitment and fail to meet enrollment targets or timelines (7–9). Although similar data is unavailable in the veterinary clinical trials space, smaller study budgets and limited personnel in veterinary trials could be associated with an even higher failure percentage.

From a scientific standpoint, robust clinical trials require an adequately powered, representative group of participants from whom data can be collected within a defined time period. This limits variables and increases the applicability and reliability of study outcomes. While the veterinary profession has adopted Russell’s replace, reduce, and refine methodology for optimizing the numbers of patients needed in research studies, patient recruitment continues to be the lynchpin of successful trial outcomes (10). Successfully enrolling an appropriate sample size prevents animals, resources, and time from being used unnecessarily either due to overpowering, which can result in wastage, or underpowering, which can lead to results that must be discarded or heavily qualified (11). Most investigators conduct power analyses as part of their study design planning because underpowered studies are of limited value to inform clinical or scientific practice. However, problems with recruitment can lead to compromises or adjustments in patient numbers, resulting in underpowered studies and a higher incidence of type two errors (11, 12). Additionally, extending the study duration to allow additional time to enroll adequate patient numbers can lead to sample degradation, personnel changes, changes in resource availability, and other variations that decrease study quality. Thus, insufficient recruitment threatens the availability of quality, evidence-based medicine through impacts on study budget, study validity, knowledge transfer, and timelines (13).

Weak recruitment can also have direct effects on the research team. For paid research personnel, funding may run out prior to study completion. For students, residents, and interns, completion of projects is often linked to degree-granting or competitive advantage in the job market and ideally should be completed during the limited training program timeframe. Failure to complete meaningful studies not only delays improvements to clinical practice but can also undermine interest in continued participation in research and result in missed opportunities, such as critical grant cycles or promotion milestones. Challenges in recruitment for trainee projects can also threaten study quality and completion as the trainee looks to move forward with their career and another individual must be found to take over the partially-completed project.

Failure to complete trials and optimize trial outcomes also fails to honor the participating animals’ and pet owners’ time and effort, which can lead to a loss of trust. A survey of owner motivations for participating in clinical trials emphasized the primacy of establishing and maintaining a relationship of trust between research institutions and pet owners (14). As the concept of “animal wastage” has broadened to include the time, biological samples, and other contributions of companion animal trial participants, studies that end prematurely or fail to reach adequate statistical power erode public trust and undermine investigators’ ethical responsibilities to animal participants and funding agencies (3, 15). Accordingly, investigators must focus on case recruitment to ensure their study’s success and the sustainability and positive impact of research.

## 3 Overcoming barriers to recruitment

### 3.1 Study design

Serious recruitment issues may arise due to flawed or unrealistic study designs, such that recruitment goals may not be achievable or the reported results may not be meaningful. Investigators must carefully balance creating a well-designed, impactful study with ensuring they have the broadest possible enrollment criteria to facilitate rapid and inclusive recruitment. Lung cancer patient advocate Jill Feldman noted during a 2022 United States Department of Health and Human Services (HHS) Office of Human Research Protections Exploratory Workshop that investigators also need to consider what patients are giving up when they agree to participate in a clinical trial and what can be done to help them participate (16). A 2019 systematic review and meta-analysis found that structural and clinical barriers inhibited trial participation for over 75% of human cancer patients (17). Onerous recheck schedules, complex and burdensome requirements, need to travel, lack of transparency, insufficient financial incentives, lack of clinical benefit for enrolled pets, and overly-strict enrollment criteria can all contribute to suboptimal recruitment.

Randomization has long been considered a cornerstone of quality clinical trials; however, concerns over placebo group assignment may impact pet owners’ willingness to participate. A survey of cat owners by Gruen et al. (14) reported that 26% of respondents would be less or much less likely to have their cat participate in a study if there was a chance of them receiving a placebo. Interestingly, a similar survey of small animal practitioners noted that 74% believed that potential assignment to a placebo group was important or extremely important to clients’ decisions about clinical trial participation for their pet (18). The use of placebo groups in clinical patients can also raise ethical considerations (19, 20). Use of real-world data or study designs that allow all patients to receive treatment (e.g., cross-over studies, standard-of-care controls, adaptive design, interim assessments, and active-controls) can be considered to help overcome these issues. Each of these options has specific implications and must be evaluated critically during study design to ensure best patient and study outcomes. For example, although “standard-of-care” controls may be most familiar in the veterinary field and initially seem most medically ethical, using them inappropriately can undermine study outcomes through generation of misleading results that are difficult to interpret, compare, and implement and waste limited research resources (21–23). A 2017 study found that 29% of human breast cancer randomized controlled trials may be using an inappropriate standard-of-care for control patients (22). Careful determination, delivery, and reporting of the “standard-of-care” used in a study, if a validated “standard-of-care” truly exists, as well as the appropriateness of this type of control in a specific study can help ensure the trial results will lead to improved patient care by guiding clinical practice recommendations and future clinical trial design (21–24).

The scarcity of funding for veterinary clinical trials can limit investigators’ ability to perform well-designed studies. Limited budgets can lead to underpowered studies because of a lack of personnel, incentives, and failure to budget sufficient funds for drop-outs, complications, or unusable data. The need to alter study enrollment criteria or design due to recruitment failure increases study variables, which can decrease study validity, particularly given the small numbers of animals typically involved in veterinary clinical research.

Excessive optimism about case enrollment numbers and timeframes is another common study recruitment problem. “Lasagna’s Law” describes the perception that the incidence of patient availability sharply decreases when a study begins (9). This dip is due to the gap between the number of theoretically eligible study participants (as identified through prevalence measures, historical case data, etc.) and participants who actually enroll. In addition to searching epidemiological or historical medical record case data, it is essential to look at the overall disease incidence, thoroughly assess the enrollment criteria, engage with relevant colleagues to assess feasibility and applicability, and realistically gauge the willingness of owners to participate in the proposed study (9). Involving diverse, representative front-line veterinary professionals and pet-owning community members early in the research process through partnerships and participation on research review boards and clinical research teams can significantly enhance recruitment. This approach increases knowledge, awareness, and trust in clinical research for care providers and pet owners. Such engagement can also help identify community priorities and reduce barriers to communication and participation during the design phase of clinical trials.

Community engagement continues to expand in human clinical research as a means of addressing inequities in access to clinical trials and healthcare in diverse populations. Although the practice is less well-established and not currently required in veterinary medicine, the latest version of the American Veterinary Medical Association (AVMA) policy on the “Establishment and use of veterinary clinical studies committees” encourages the inclusion of “at least one person not affiliated with the entity performing the study” (25). Additionally, tracking the reasons for failure to enroll eligible animals can provide important insights to improve study design.

Recently, increased attention has been given to improving the scientific rigor of veterinary clinical study design as well as the reporting of clinical trial outcomes. Such initiatives aim to produce quality, transparent, and reproducible data that informs evidence-based clinical decision-making. Poor study design may result in a significant loss of time and resources and generate outcome assessments and noninformative comparisons that are clinically irrelevant, unnecessary, and potentially misleading (12). Resources such as the PetSORT Guidelines, a veterinary-focused adaptation of the Consolidated Standards of Reporting Trials (CONSORT), seek to create standard reporting guidelines that are harmonized and evidence- and consensus-based (3, 26, 27). The PetSORT checklist of items to address in a clinical trial report are useful for investigators to incorporate best practices in both study design and study reporting and for editors, reviewers, and content users to assess publications (3). With specific attention to recruitment, a simplified version of this checklist may be helpful to ensure that practical considerations are well-balanced with scientific rigor (Table 1).

**Table 1:** Recruitment best practices checklist.

**Planning phase:** Consider appropriate study groups and sample size Placebo versus standard-of-care versus real world data Randomization scheme Assess enrollment criteria to determine if it can be broadened Examine potential barriers to anticipated case numbers and timeline that are drawn from prevalence measures and/or historical case data Consider feasibility related to logistical and socio-economic barriers *from owner perspective* Physical access Travel requirements Time investment Language/comprehension Impact on human-animal bond Outcome measures meaningful to owners Other Consider feasibility and barriers *from a patient/pet perspective* Well-being/stress Clinical benefit Other Ensure appropriate financial incentives are in place Create a detailed study communication plan Review PetSORT and other study guidelines to help ensure that all elements of the study have been considered Consult lay reviewer/patient/pet/owner advocate as needed **Conduct phase:** Create advertising materials that are clear and understandable Utilize “Mix and Match Recruitment Strategies (see Figure 2)” to determine optimal advertising plan Ensure transparency regarding financial incentives and study costs Track reasons for enrollment failures to inform future study designs Consider whether barriers to participation can be removed Use a single point-of-contact, if possible, for owner inquiries, recheck scheduling, etc. Maintain ongoing communication within the study team Regular meetings/check-ins Shared up-to-date communication platform Discuss challenges and solutions if problems arise Maintain ongoing communication *with owners* Newsletters/E-newsletters Enrollment updates Study-related content Thank-you cards and/or branded items such as bandanas, keychains, etc. Maintain ongoing communication *with primary care veterinarians* Newsletters/E-newsletters Patient summaries Thank-you cards **Dissemination phase:** Conduct study completion meeting with study team Disseminate lay summary of results to participating owners, funding agencies, and referring veterinarians Consider both scientific and lay publications/news outlets Use PetSORT guidelines to ensure complete reporting of study results Identify additional opportunities for dissemination targeting affected populations

### 3.2 Logistics

Failure to comprehensively assess study logistics from the enrolled pet and owner’s perspective can limit recruitment. In human medicine, patient-centered clinical trials are becoming more common. When the public engages in medical research, it strengthens researcher accountability, increases communication and transparency, and may identify priorities and concerns not initially recognized by the researchers (28, 29). Stakeholder engagement has been incorporated into human clinical trials to address this issue. In 2018, the Clinical Trials Transformation Initiative (CTTI) proposed a framework for improving clinical trials recruitment emphasizing “human factors” as a key means of running more efficient trials. The framework encourages investigators to consider barriers such as physical access, time for appointments, and language difficulties, among others, during study design (4, 15, 30, 31).

Veterinary clinical trials require the pet owner to facilitate their pet’s participation, and therefore similar practices should be encouraged in the veterinary context. Practicalities should be weighed against the ideal study design. For example, when running a trial from a university research center, investigators’ recruitment efforts will likely benefit from the institution’s prestige. However, accessibility of the study site may influence enrollment and continued participation such that investigators may want to consider the possibility of having rechecks take place at the pet’s primary care practice. Although partnering with other institutions and hospitals comes with challenges, potential benefits can be seen in increased owner compliance, faster recruitment, and improved study power and reproducibility. Traditional offline recruitment strategies, such as soliciting in-clinic cases or clients within a short radius of the clinic, may be more convenient for the investigators. However, the limited scope of such strategies may create bias and limit the diversity of study participants and the generalizability of the study outcomes (7). Adapting clinical trial design to real-world circumstances may require larger participant numbers but has the potential to make study results more applicable to the intended population.

### 3.3 Representative participation

The socio-demographic characteristics of the pet owners should be considered when developing recruitment strategies to decrease bias and improve study outcomes and generalizability. Owners of sick pets incur increased costs associated with managing their pets’ health conditions. Some elements of a study’s design can inadvertently limit enrollment and fulfillment of study requirements by increasing the burden of pet owners. For example, if participation requires additional financial commitment in the form of missed work, travel, parking, pet care, etc., the subset of owners who are willing and able to participate shrinks – a demonstration of Lasagna’s Law in action (9). Investigators might end up only enrolling pets who benefit in other ways from their owners’ higher socio-demographic status, which could alter the study results by reducing the likelihood of enrolling a representative sample population.

The influence of lack of diversity and inclusion on clinical trial outcomes has been studied in human medicine and is an identified priority. Guidelines by CONSORT and PetSORT encourage the identification and tracking of reasons for refusals and loss to follow-up, which allows researchers to examine where skewed selection of participants or drop-outs due to socio-demographic concerns may arise (3, 4). Failure to include a diverse population can negatively impact the applicability of the research results. In human medicine, dose-finding studies often fail to include sufficient numbers of women despite their variable pharmacokinetics, body weight, and higher prevalence of medication use, and many post-approval drug withdrawals in the human market have been due to toxicity in women (32, 33). Biological sex is only one of many study participant variables (e.g., breed, species, age, comorbidities, exposures) that should be considered in optimal study design, particularly as knowledge advances in the area of precision medicine.

### 3.4 Incentives for participation

Clarity concerning study costs and obligations is critical to successful study recruitment. However, financial conversations are challenging and may be considered a lower priority by both the investigators and owners during complicated study enrollment discussions (16). Estimates of owners’ out-of-pocket costs may be missing from study descriptions or vague (e.g., “study-related costs are covered”).

Although more straightforward for a layperson to understand compared to medical terminology and procedures, the implications associated with incentives can be complex. Incentives can come in multiple forms, including reimbursement for costs incurred by study participants (i.e., parking, mileage), compensation for participants’ time, tokens of appreciation for study participation, as a part of the experimental intervention (i.e., studies investigating the influence of money on preferences and actions), and structured payments to encourage continued participation in the study. Studies might cover the costs of diagnostic testing, appointments, and other aspects of standard care, the cost of the study intervention, and allow access to novel treatments for participants.

Incentive payments involve many challenging issues, from ethical, theoretical, and psychological concepts, to increased financial, tax, and administrative burden. Research studies and scholarly discussion continue to question whether incentive payments undermine studies’ scientific validity and social value by biasing participants’ decision-making and compromising informed consent. An alternative view holds that incentives improve study conduct, inclusivity, and social value (34). Human clinical trial participants may find studies where compensation was not provided to be disrespectful of their time and effort, and some participants judge gift cards and other non-cash incentives to be patronizing and inadequate (16). However, incentives can also deter study recruitment if they are interpreted to undermine a participant’s contribution (e.g., participation is viewed as transactional instead of altruistic) or to be indicative of the risk of the study (35–37). Careful consideration is needed to find the best ways to incorporate incentives into clinical trial design and conduct until additional evidence-based recommendations are available.

The literature about incentive payments in veterinary clinical trials is sparse. Gruen et al. (14, 18) reported that over 73% of surveyed cat owners and 87% of surveyed small animal practitioners ranked “free services” (e.g., laboratory tests, radiographs, examinations, etc.) as the “most encouraging” or “best” owner incentive to participate with their pet in a clinical trial. Current estimates in the United States and the United Kingdom put the percentage of insured pets between 1–22%, depending on location and species (38–40). Given the lack of insurance coverage and the growing role of pets as companions and family members, the offer of free healthcare services may be especially powerful in the veterinary context. The tax implications of cash incentives may also play a role in these preferences.

**Figure 1:**
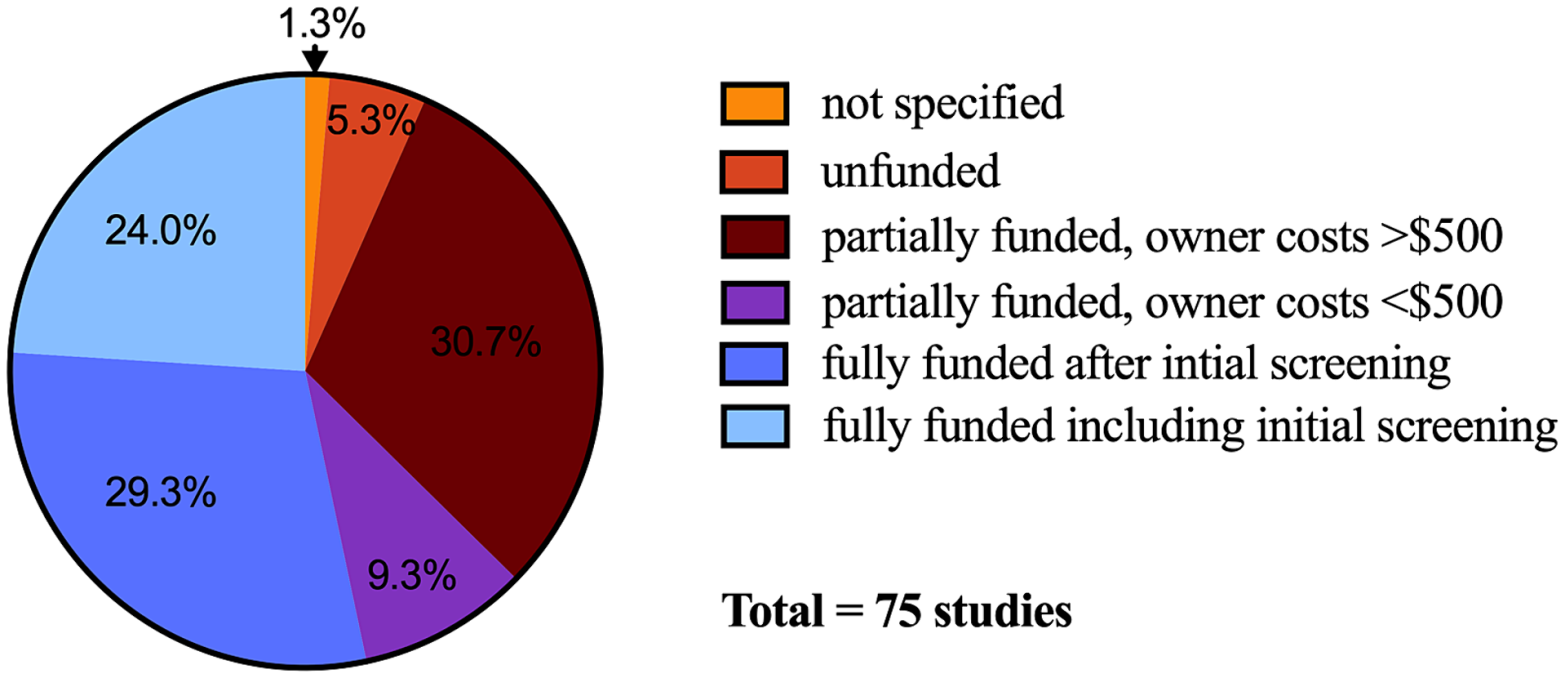
Financial incentive categorical choices in United States Dollars selected for veterinary clinical trials (*n* = 75 studies) posted on the American Veterinary Medical Association Animal Health Studies Database. Data collected between November 17 and December 17, 2023. Not specified, *n* = 1; unfunded, *n* = 4; partially funded, owner costs <$500, *n* = 7; partially funded, owner costs >$500, *n* = 23; fully funded after initial screening, *n* = 22; fully funded including initial screening, *n* = 18.

In practice, robust financial incentives are likely uncommon in veterinary clinical trials. Evaluating 75 actively enrolling veterinary clinical studies listed on the American Veterinary Medical Association’s (AVMA) Animal Health Studies Database (AAHSD) in November and December of 2023, only 24% (18/75) of studies were listed as fully funded including initial screening (Figure 1) (41). Even when a trial is fully-funded, the process of obtaining a diagnosis is often lengthy and expensive, and owner out of pocket costs were listed as greater than $500 *after* enrollment in 31% (23/75) of the evaluated studies. Substantial out-of-pocket costs can limit study enrollment, compliance, and retention.

In recent years the discussion around payment for research participation in human clinical trials has incorporated the idea of justice. Clinical research requires participants, and therefore compensating participants, or in the case of veterinary medicine, their owners, as part of the research team for their time and contributions can be considered both ethical and necessary (16). Payment can remove the burden of participation and improve equity and representativeness in clinical trials. In addition, providing payment demonstrates respect for patient or pet owner time, expertise, risk, and value to the research process, and can remove power imbalances and improve commitment in study conduct. Halpern et al. (37) found that although higher payments may motivate research participation, commonly used payment levels were not considered bribes or unethical.

Researchers and funding agencies must carefully consider what incentive to provide for research to achieve successful patient recruitment numbers and best study outcomes, and protocol review boards must decide if study incentives are ethical. Although funding is often limited in veterinary research, providing compensation for research participation has the potential to improve recruitment and study outcomes and acknowledges the scientific and societal benefit provided by the owner and their pet, particularly considering the potential translational applications of many veterinary clinical trials. In all cases, compensation details, including what is being provided and by whom, must be transparent and consistent, and can thereby engender trust in the research process.

### 3.5 Personnel resources

A positive (or negative) experience with the primary study communicator can impact patient recruitment and retention. In human clinical trials, nurses and data managers contribute more than 60% of the workload in clinical research compared to 9% from physicians (42). In a human prostate cancer trial comparing the effectiveness of the primary communicator (nurses vs. surgeons), nurses tended to spend longer on each recruitment, were as effective in recruitment as surgeons, and, ultimately, were more cost-effective (43).

Information on the distribution of workload in recruitment for veterinary studies is sparse. However, the primary responsibility for contact and study conduct in veterinary studies often falls on the study clinician; this responsibility may shift to the research coordinator or technician if available. Dedicated clinical trial units in veterinary medicine are often small, if they exist at all. Recent survey data reported that just over half (59.1%) of the 22 responding veterinary academic institutions across the United States and Canada had a centralized veterinary clinical research unit, which employed a median of approximately 4 full time equivalent personnel (44).

When available, clinical trials/research teams may vary by study type, study purpose, and between study sites. Stable, permanently-funded positions allow flexibility to adapt to the changing needs of the clinical trials program. All study personnel should be trained in their respective duties (i.e., consenting, study procedures, adverse event reporting, etc.). Although formal certification in veterinary clinical trials is not currently available, resources are expanding and training should be encouraged where available (Supplementary Table). Use of human clinical trials training resources is an option, although there are limitations in the applicability of some information, such as regulatory requirements. Expanding training for areas such as recruitment, consenting, and adverse event reporting to all hospital staff, students, and trainees can help ensure all individuals are supporting quality clinical research and consistent communication with pet owners. Specialty training in sensitive situations, such as trials involving emergency medicine cases, may also be beneficial for successful recruitment.

With small teams and limited resources, the future of veterinary clinical trials may involve a shift to expanded online recruitment strategies. In a systematic review and meta-analysis of human clinical trials, online recruitment has been reported to offer superior time efficiency and cost-effectiveness compared to offline (in-person) recruitment (7). There is a fine balance, however, as this study also reported that in-person recruitment had a higher conversion rate when compared to online (7).

### 3.6 Advertising

**Figure 2:**
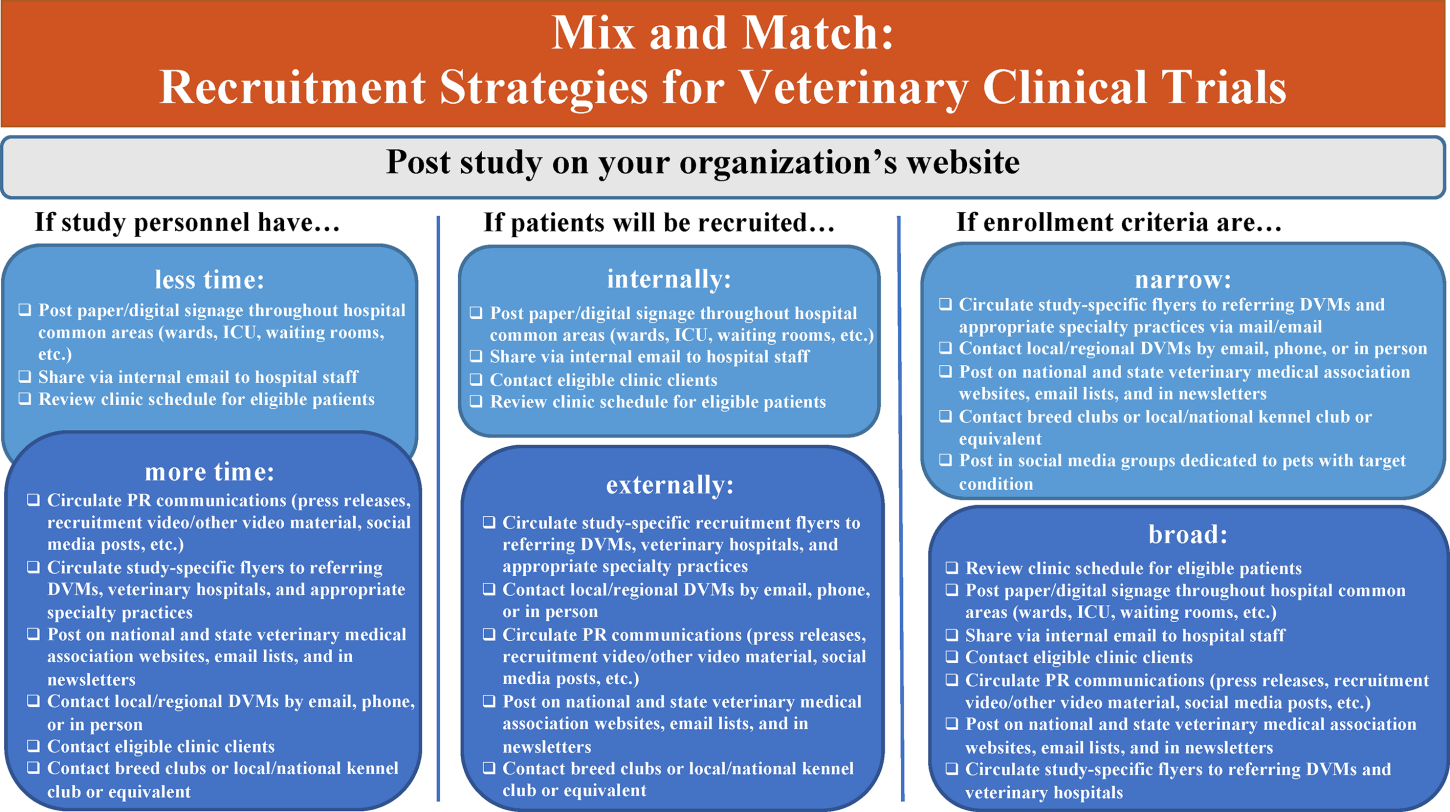
Recruitment strategies for veterinary clinical trials. ICU, Intensive Care Unit; PR, Public Relations; DVM, Doctor of Veterinary Medicine.

Another major impediment to successful recruitment is study advertising. When possible, the clinical trials team can create recruitment materials. However, teams may have limited capacity due to personnel resources and a lack of training in effective advertising and marketing strategies. Including individuals with expertise in communication and with the target communities in the clinical trials team can help optimize advertising materials and strategies. If such expertise is unavailable, developing a knowledge bank of available, successful advertising and marketing strategies can be useful. Strategies can be mixed and matched to suit specific project needs and resources (Figure 2).

Even among veterinarians, there is a lack of awareness of the availability and impact of clinical trials in veterinary medicine. A 2017 survey (18) found that 28% of veterinarians did not usually learn about clinical trials in their area. Even recent DVM graduates from academic institutions were often unaware of clinical research studies, and only 55% of recent graduates had investigated clinical trial participation for their patients (18). Veterinarians were more likely to recommend clinical trial participation if a respected investigator was conducting the study, an academic institution sponsored the study, and the study results were disseminated back to the veterinarian and scientific community (18). A 2014 survey of cat owners reported that the majority (75–89%) viewed veterinarian recommendation as a significant factor in study participation (14). When asked which method of recruitment communication is the most effective, referring veterinarians preferred email communication to the practice (70%) followed by clinical trials websites (60%), printed materials sent to the practice (54%), and visits from the study investigator (43%) (18).

Although referring veterinarians play an important role in clinical trials recruitment, successful advertising strategies should also target pet owners. Data from The Ohio State University’s Clinical Trials Office show that the vast majority (96%; 1,226 of 1,278 total forms) of initial screening forms completed for ongoing veterinary clinical trials from 2018–2021 were by pet owners (personal communication). Similarly, efforts in recruitment to human clinical trials have shown that directing outreach toward potential study participants and their caregivers was more successful and cost-effective than targeting primary care health professionals (45).

Ultimately, recruitment materials should empower pet owners to “read, understand, and act” on their pet’s behalf by providing informed consent for study participation (46). Previous studies have explored issues around the readability of study materials (2, 46, 47). Most study-related materials far exceed the average American’s 8th-grade reading level and the recommended 6th-grade reading level for medical information (2, 46, 47). Failure to provide clear materials can cause issues with both recruitment and compliance by compromising the ability of pet owners to make informed medical decisions and potentially eroding public trust in veterinary professionals (46, 48). Simple remedies, such as readability calculators (e.g., Microsoft Word and the free, web-based readability tool Automatic Readability Checker) (46) and artificial intelligence (AI)-based writing tools (e.g., ChatGPT and others), are available to help improve readability. Employing the assistance of a patient/pet owner advocate or a lay reader are alternative strategies to ensure that written materials are understandable and appealing to the target audience. Additionally, using graphics and other visuals (e.g., bullet points, videos, study visit calendars) with easy-to-read study information, including study personnel contact information, can help overcome literacy and numeracy barriers to study recruitment (16, 46). In all cases, and particularly with the use of new AI tools, users must critically review the information to ensure accuracy and that ethical issues such as potential bias, privacy, and authorship are addressed (49).

Efforts have been made to create a centralized, easily-searchable registry of clinical trials to increase awareness of ongoing veterinary clinical trials. The American Veterinary Medicine Association’s (AVMA) Animal Health Studies Database (AAHSD) was launched in 2016 to help connect pet owners and referring veterinarians with reliable and comprehensive study listings (41, 50). The available development and implementation resources had limited the utility of the database, however, the AVMA launched an updated database, the AVMA Veterinary Clinical Trials Registry, in 2024 intended to address previous limitations.

### 3.7 Retention

Study attrition refers to the failure to retain participants after enrollment, which can occur at any time during the study and for various reasons depending on the population, study duration, condition, intervention, and outcome measures used (51). Attrition rates of 26% for the primary endpoint and 44% for the end of the study have been reported in human supportive and palliative oncology clinical trials (52). Some studies have shown attrition rates up to 67% (53). Loss of this number of patients, particularly from one patient group, can lead to sampling bias and/or reduction in sample size and affect the internal and external validity of studies. Reasons for attrition in the above-mentioned studies included patient characteristics, such as high baseline symptom burden, and study characteristics including placement in a placebo group, longer study duration, and outpatient studies (52, 53). Difficulty in understanding the study requirements and consent form, failure to obtain timely responses from study personnel, communication failures, socioeconomic conditions, and experiencing stress during study visits are other commonly-cited causes of attrition (54, 55). Participant retention is important for all studies, especially those with longitudinal designs, and recording reasons for patient dropout can provide information to improve future study recruitment and conduct (55, 56).

Retention in veterinary clinical trials relies on a solid and consistent veterinary client-patient relationship (VCPR) centered around communication from the first contact (i.e., study recruitment), and throughout the study, up to and following dissemination of study results. Clinical trials are often conducted at a specialty referral hospital; therefore, this referral can be viewed as an extension of a primary veterinarian’s care (57). The VCPR extends between study veterinarians, their clients (and patients), collaborating colleagues, and the veterinary profession. Continuation of care is an important concept in study design and recruitment, both to ensure patient support continues after a study ends, as well as to overcome a potential barrier to referral—the fear of losing patients through clinical trial participation (18, 45).

For clinical trials, streamlined communications throughout the study process facilitate recruitment, repeated visits, and adherence to strict study protocols. In an Australian longitudinal water quality study, families who continued to participate for the 68-week study duration cited being kept well-informed via a monthly newsletter as the strongest determining factor of their long-term compliance (58). Similarly, pet owners are 40% more likely to comply with veterinary recommendations when communication is clear, thorough, and trustworthy (59).

Having a single contact person or designated care team can help create continuity and trusted relationships. In addition, simple gestures can help to maintain this relationship, such as sharing study results once published or larger community outreach events such as study days or celebrations (45). Exposure to branded promotional items may also increase awareness of and affinity for the clinical research enterprise, especially when the “brand” is unfamiliar or for longitudinal studies where retention can present special challenges (60). Chhatre et al. (61) demonstrated success in recruiting and retaining participants in a multi-site human prostate cancer study by using a multi-faceted, patient-centered strategy that included personalized thank-you notes, contact via preferred communication methods, and providing study progress reports to enrolled patients. Such efforts can help create a community of individuals supportive of veterinary clinical research that can facilitate future recruitment and retention efforts across the profession. With the rise of social media, increased owner access to electronic veterinary medical records, and other digital tools, there has been an increase in direct outreach to potential study participants via digital strategies (46, 51). While social media has the potential for unwanted attention on research if there are adverse outcomes, an owner’s high investment in studies of specific diseases, breeds, and/or treatment(s) can further increase awareness and advertisement of the study. A positive clinical trial experience can also influence pet owners’ future study participation and support.

## 4 Conclusions and future directions

Clinical trials are performed to improve patient health outcomes, and a successful clinical trial requires adequate numbers of participants. However, many factors impede effective study recruitment, and there is a collective need to share and implement current best practices for recruitment, including study design and logistics, encouraging representative participation, use of incentives and personnel resources, and strategies for advertising and participant retention. Although the use of human clinical trial data and resources can provide some guidance, evaluation of current practices and opportunities for process improvement in the conduct of veterinary clinical trials is needed.

Greater awareness of veterinary clinical trials and standardization of study conduct can create opportunities for expanded and effective collaborations. According to a 2021 survey of North American veterinary schools, 80% of veterinary clinical studies were conducted at a single site (44). While multi-center studies require a larger up-front time investment, human multi-center clinical trials have demonstrated the benefits of a larger and more equitable recruitment pool, and increased study efficiency may outweigh the costs. Efforts are underway to address challenges with multi-center trials in veterinary medicine. For example, the development of a veterinary Streamlined, Multisite, Accelerated Resources for Trials (SMART) Institutional Animal Care and Use Committee (IACUC) reliance platform similar to the human SMART Institutional Review Board (IRB) may help facilitate larger collaborative studies (62, 63). Ultimately, collaboration can reduce redundancy and support more robust and consistent study design, efficient ethical review, study advertising and recruitment, good clinical practices, and pooling of resources to run studies that lead to improved clinical decision making for veterinary patients.

Clear communication between all stakeholders throughout the research process is key to successful and meaningful clinical research, and training in science communication can be beneficial to all members of a research team. Increased community engagement in veterinary clinical research, including pet owners, advocates, and primary care veterinarians and staff, is another area of opportunity to foster support for research and improve the research process. Investment in trained, dedicated clinical trial personnel and infrastructure can support successful communication and collaboration and drive improvements in veterinary clinical medicine by providing the time and expertise to effectively complete impactful clinical trials. Continued development of collaborations surrounding best practices and training opportunities in veterinary clinical research will improve the impact of veterinary clinical trials teams, while also promoting workforce development and alternate career paths for veterinary professionals. Including plans for widespread dissemination and implementation of veterinary clinical research results will validate the research process, honor the participants, maximize the positive impact on clinical practice, and support future successful research efforts.

Ultimately, putting recruitment at the forefront of clinical trials can minimize patient and pet owner burden and maximize patient and pet owner appreciation, all while achieving sound scientific and clinically relevant results. Given the diversity of veterinary clinical trials, a one-size-fits-all approach to study recruitment will never exist. However, researchers can thoughtfully draw from an expanding variety of evidence-based, patient-centered approaches to improve their odds of successful study recruitment and completion.

## Author contributions

MQ: Writing – original draft, Writing – review & editing. CM: Writing – original draft, Writing – review & editing. TW: Conceptualization, Funding acquisition, Writing – original draft, Writing – review & editing.
