# Supplementary material for: Best practices for recruitment in veterinary clinical trials
Source: Front Vet Sci. 2024 Jul 17;11:1418747. doi: 10.3389/fvets.2024.1418747 (PMC11288851; doi:10.3389/fvets.2024.1418747)
Supplement: Supplementary file 1 [file Table_1.docx]

Supplementary Material

**Supplemental Table 1: Veterinary Clinical Trials Resources***

| **Training** | **Website/Contact** |
| --- | --- |
| COHA Clinician Scientist Training Workshop | <https://www.ctsaonehealthalliance.org/resources/education-training/> |
| COHA Translational Research Immersion Program (TRIP) | https://www.ctsaonehealthalliance.org/resources/coha-translational-research-immersion-program-trip |
| Good Clinical Practice for Veterinary Clinical Trials Module | https://ilearn.tuftsctsi.org/product?catalog=OH102_2019_Online |
| Client Consent: Best Practices for Veterinary Clinical Trials | https://ilearn.tuftsctsi.org/product?catalog=OH2021_01_Online |
| Risk Assessment and Management in Veterinary Clinical Trials | https://ilearn.tuftsctsi.org/product?catalog=RC2TU_2023_04 |
| Regulatory Aspects of Veterinary Clinical Trials | *in development* (Tufts CTSI I LEARN platform) |
| **Meetings/Continuing Education** | **Website/Contact** |
| Best Practices in Clinical Research Conference | https://vet.uga.edu/event/best-practices-in-clinical-research/ |
| **Clinical Trials Websites** | **Website/Contact** |
| AVMA Clinical Trials Registry | https://veterinaryclinicaltrials.org |
| Veterinary Cancer Society | http://vetcancersociety.org/pet-owners/clinical-trials/ |
| FDA CVM Clinical Field Studies for Animal Cells, Tissues, and Cell- and Tissue-Based Products (ACTPs) | https://www.fda.gov/animal-veterinary/cell-and-tissue-products-animals/clinical-field-studies-animal-cells-tissues-and-cell-and-tissue-based-products-actps |
| **Research Collaborations** | **Website/Contact** |
| SMART IACUC | https://ctsaonehealthalliance.org/2023/09/27/smart-iacuc-network/ |
| ACVIM Research Colloquiums | https://www.acvim.org/research/research-colloquiums |

*Accessed May 18, 2024. COHA = Clinical and Translational Science Award One Health Alliance; CTSI = Clinical and Translational Science Institute; AVMA = American Veterinary Medical Association; FDA CVM = United States Food and Drug Administration Center for Veterinary Medicine; SMART IACUC = Streamlined, Multisite, Accelerated Resources for Trials Institutional Animal Care and Use Committee; ACVIM = American College of Veterinary Internal Medicine
